# Benchmark Comparison of Bone Marrow Aspiration Systems: Increased Progenitor Cell Concentrations Achieved Without Centrifugation Using a Novel Filtration Design With High Surface Area

**DOI:** 10.7759/cureus.110747

**Published:** 2026-06-12

**Authors:** Anthony Scillia, Teo Mendez, Dan Kuebler, Bob Salvat

**Affiliations:** 1 Sports Medicine/Orthopedics, Seton Hall University, Paterson, USA; 2 Sports Medicine, Lenox Hill Hospital, New York, USA; 3 Department of Biology, Franciscan University of Steubenville, Steubenville, USA; 4 Department of Clinical Research, SurGenTec, LLC, Boca Raton, USA

**Keywords:** bmac, bone marrow aspirate, bone marrow concentrate, centrifugation, colony-forming unit-fibroblast, hemodilution, mesenchymal stem cells, orthobiologics, progenitor cells, regenerative medicine

## Abstract

Background

Bone marrow aspirate (BMA) is widely used in orthopedic and regenerative procedures due to its content of mesenchymal stromal cells (MSCs), hematopoietic progenitors, and supportive cellular elements that contribute to bone healing and tissue repair. Conventional bone marrow aspiration techniques are limited by progressive hemodilution, in which peripheral blood increasingly contaminates the aspirate as volume is drawn from a fixed location. This process both reduces MSC and progenitor concentrations and increases variability in aspirate samples.

Objectives

The purpose of this study was to characterize the cell yield of a novel bone marrow aspiration system (B-MAN™ Bone Marrow Aspiration System, SurGenTec, Boca Raton, FL) under standardized testing conditions. These results were compared with published benchmark data from a study that analyzed centrifuge-processed bone marrow concentrate (BMAC) systems and a no-spin aspiration device analyzed in the same reference laboratory used in this study.

Methods

A retrospective analysis was performed on bone marrow aspirate obtained from the anterior iliac crest of five adult male subjects (age range 18-44) using the B-MAN™ system during elective orthopedic procedures. Procedures included anterior cruciate ligament reconstruction, hip labral repair, anterior cruciate ligament* *(ACL) reconstruction with tibial plateau fixation, and Achilles tendon repair. Aspirations of 1 mL were performed using a 10 mL syringe through a single cortical access site. Samples were not centrifuged. Laboratory analysis of a portion of the aspirate included total nucleated cell (TNC) concentration, CD34+ cell count, colony-forming unit-fibroblast (CFU-f) concentration, and cell viability.

Results

Mean counts for these aspirates were 102 × 10⁶ TNC/mL, 15,145 CFU-f/mL, and 1,143,216 CD34+ cells/mL, with a mean viability of 96.9%. The mean of individual CFU-f:TNC ratios was 0.013% (range 0.006-0.020%), at or slightly above the upper bound of the expected range for minimally diluted marrow (0.001-0.01%), consistent with known inter-subject variability in marrow cellularity. These values place B-MAN aspirates at the upper range of concentrations reported in published benchmark studies evaluating centrifuge-processed BMAC and optimized no-spin aspiration systems.

Conclusions

In this descriptive laboratory evaluation, aspiration using the B-MAN™ system yielded high progenitor cell concentrations in unprocessed marrow aspirate. These findings support further investigation of aspiration interface engineering as a complementary and potentially impactful strategy to needle tip relocation for reducing hemodilution.

## Introduction

Bone marrow aspirate (BMA) has become an increasingly important treatment component in orthopedic and spine surgery, particularly in the context of biologic augmentation for regenerative sports medicine, spinal fusion, and general orthopedic applications [1-6]. Autologous BMA is widely used as a source of osteogenic and immunomodulatory elements, either delivered in its native form or following processing through concentration systems. The rationale for these applications rests on the well-established multipotency of marrow-derived mesenchymal stromal cells, which retain the capacity to differentiate along osteogenic, chondrogenic, and adipogenic lineages [7]. As the use of biologics continues to expand across indications, the quality and composition of the aspirate have emerged as key determinants of therapeutic potential, since the cellular composition of the aspirate plays a critical role in achieving successful tissue repair [8-10]. 

Bone marrow is a highly complex biological environment composed not only of mesenchymal stromal cells and hematopoietic progenitors, but also endothelial progenitor cells, immunoregulatory cells, cytokines, and extracellular vesicles [7,10-14]. These components interact within a dynamic signaling network that contributes to tissue repair through both direct cellular differentiation and paracrine effects. Processing techniques that selectively concentrate certain fractions may alter this balance, potentially removing or diminishing components that contribute to the overall therapeutic effect [14]. Beyond processing, the mechanics of aspiration itself, including the fluid-dynamic behavior of marrow at the needle interface, also influence the cellular yield ultimately delivered to the patient.

The cellular metrics of aspirate quality

The biologic quality of BMA is typically characterized using cellular metrics such as total nucleated cells (TNC), cluster of differentiation (CD)34-positive (CD34+) hematopoietic progenitor cells, and colony-forming unit-fibroblasts (CFU-f), a functional surrogate for mesenchymal stromal/stem cells. These metrics represent distinct biological compartments within the marrow microenvironment. TNC reflects the overall cellular population, including immune and stromal elements; CD34+ cells indicate hematopoietic and endothelial progenitor activity; and CFU-f reflects adherent stromal progenitors capable of tri-lineage differentiation and paracrine signaling [7]. The relative balance of these populations is critical, as high total cell counts alone do not necessarily indicate a marrow-rich aspirate if peripheral blood contamination is present [3,8,9].

Clinical evidence supports the importance of progenitor cell content in determining therapeutic efficacy. Hernigou and colleagues demonstrated a direct relationship between CFU-f counts and successful healing in nonunion cases, with lower progenitor cell numbers associated with delayed union or treatment failure [4]. Chaput and colleagues demonstrated a correlation between mesenchymal stromal cells (MSCs) and other bone marrow-derived cellular components in the healing rates in anterior cervical discectomy and fusions (ACDF’s) [5]. These findings emphasize that both the concentration and the absolute number of progenitor cells delivered are clinically relevant, reinforcing the importance of optimizing aspirate quality at the time of harvest rather than relying solely on post-processing techniques.

The challenge of hemodilution

Despite its clinical utility, obtaining a high-quality marrow aspirate remains technically challenging. A primary limitation is peripheral blood contamination, or hemodilution, which occurs rapidly during aspiration. Clinically, this is observed as a transition from a viscous, marrow-rich aspirate in the initial milliliters to a progressively thinner, blood-dominant sample with continued aspiration. Hemodilution reduces both the relative and absolute concentration of progenitor cells, particularly CFU-f, thereby diminishing the biologic potency of the aspirate [1,9]. This challenge has historically driven the adoption of centrifugation-based concentration systems intended to recover progenitor populations from diluted aspirates [5,14-16]. However, this approach introduces several limitations. It typically requires large initial aspirate volumes, often in the range of 60-120 mL, which may increase procedural time, blood loss, and patient morbidity. Additionally, centrifugation may result in the loss or alteration of biologically relevant components, including cytokines, extracellular vesicles, and certain cell populations [17].

The aspiration technique has been shown to significantly influence hemodilution. Multiple studies have demonstrated that small-volume aspirations, typically 1-2 mL per site, yield higher concentrations of progenitor cells compared to larger-volume draws [1,2,9]. Larger aspirates increase the likelihood of sinusoidal disruption and accelerate peripheral blood ingress. Similarly, syringe size and aspiration dynamics influence pressure gradients, with smaller syringes and rapid plunger displacement producing higher-quality aspirates [2]. Rapid creation of negative pressure appears to preferentially mobilize marrow before peripheral blood can enter the aspiration field, further supporting the importance of controlled aspiration mechanics.

Limitations of conventional needle design and centrifuge-based processing

The design of conventional bone marrow aspiration needles, derived from Jamshidi-type devices, has remained largely unchanged for decades, relying on circular side fenestrations that create localized regions of high-velocity inflow and shear sufficient to disrupt sinusoidal structures and accelerate peripheral blood contamination [12,13]. In contrast, modifying the geometry of the aspiration interface to increase total intake surface area may alter these flow dynamics. Distributing suction across a larger area reduces local velocity and shear stress, potentially preserving sinusoidal integrity and limiting the rapid influx of peripheral blood. Thin elongated intake geometries, for example, increase surface area and distribute negative pressure more evenly, which may reduce turbulence and create a more laminar flow profile during aspiration. This approach has been proposed to preferentially mobilize marrow while minimizing vascular disruption and subsequent hemodilution.

Taken together, these considerations suggest that improving the quality of bone marrow aspirate at the time of collection may represent a more effective strategy than relying on downstream concentration. If aspiration techniques and device design can consistently yield marrow-rich aspirates with high progenitor cell content and minimal peripheral blood contamination, it may be possible to achieve biologically effective cell delivery without the need for centrifugation, particularly given the limitations of centrifugation described above.

The present study evaluates the novel B-MAN™ Bone Marrow Aspiration System, designed to address these limitations by modifying the aspiration interface itself. The study involved the examination of total nucleated cell (TNC) concentration, CD34+ cell count, colony-forming unit-fibroblast (CFU-f) concentration, and cell viability in aspirates harvested using the B-MAN™ device.

## Materials and methods

Study design

This investigation was designed as a laboratory evaluation to characterize cell yield metrics of bone marrow aspirate obtained using the B-MAN™ system. The study was not designed to assess clinical outcomes or establish clinical efficacy [3-5]. The objectives of this investigation were to (i) characterize total nucleated cell (TNC), CFU-f, and CD34+ concentrations, and cell viability, in unprocessed bone marrow aspirate obtained using the B-MAN™ system, and (ii) descriptively contextualize these values against published benchmark data analyzed at the same reference laboratory.

This investigation was a retrospective, non-interventional laboratory characterization of bone marrow aspirate cellularity; participants were not assigned to a protocol-driven intervention, and no clinical outcomes were measured. As such, the study does not meet the ICMJE criteria for prospective clinical trial registration. The study was approved by the Franciscan University Institutional Review Board (Protocol #2020-1), and informed consent was obtained from all participants prior to aspiration.

Subjects and aspiration procedure

The bone marrow aspirate was obtained from five adult male subjects (age range 18-44) undergoing elective orthopedic procedures, including anterior cruciate ligament reconstruction (n = 2), hip labral repair (n = 1), ACL reconstruction with tibial plateau fixation (n = 1), and Achilles tendon repair (n = 1) (Table 1). This case mix reflects the broad applicability of autologous bone marrow preparations across orthopedic indications [18]. Aspirations of 1 mL were performed from the anterior iliac crest, which has been shown to provide reliable progenitor cell yields [11]. A single cortical access was established to gain access to the marrow space, and bone marrow aspirate was drawn, consistent with evidence that single-site aspiration techniques can achieve aspirate quality comparable to multi-site approaches while reducing patient discomfort [19]. No post-harvest centrifugation or concentration was performed, consistent with a workflow designed to minimize intraoperative processing time and its associated operating room costs [20].

Aspiration technique

Following standard sterile preparation and draping of the anterior iliac crest, the B-MAN™ needle, with the relocation sleeve preset to expose 2 cm of cannula beyond the bone stop, was advanced through the subcutaneous tissues until the trocar tip contacted the cortex. The cortex was then breached, and the needle was advanced until the bone stop was seated against the iliac crest, and 1 mL of marrow was drawn into a 10 mL syringe through a single cortical access. The sample was transferred to a collection test tube and shipped on ice to the reference laboratory for analysis.

B-MAN™ bone marrow aspiration system

**Figure 1:**
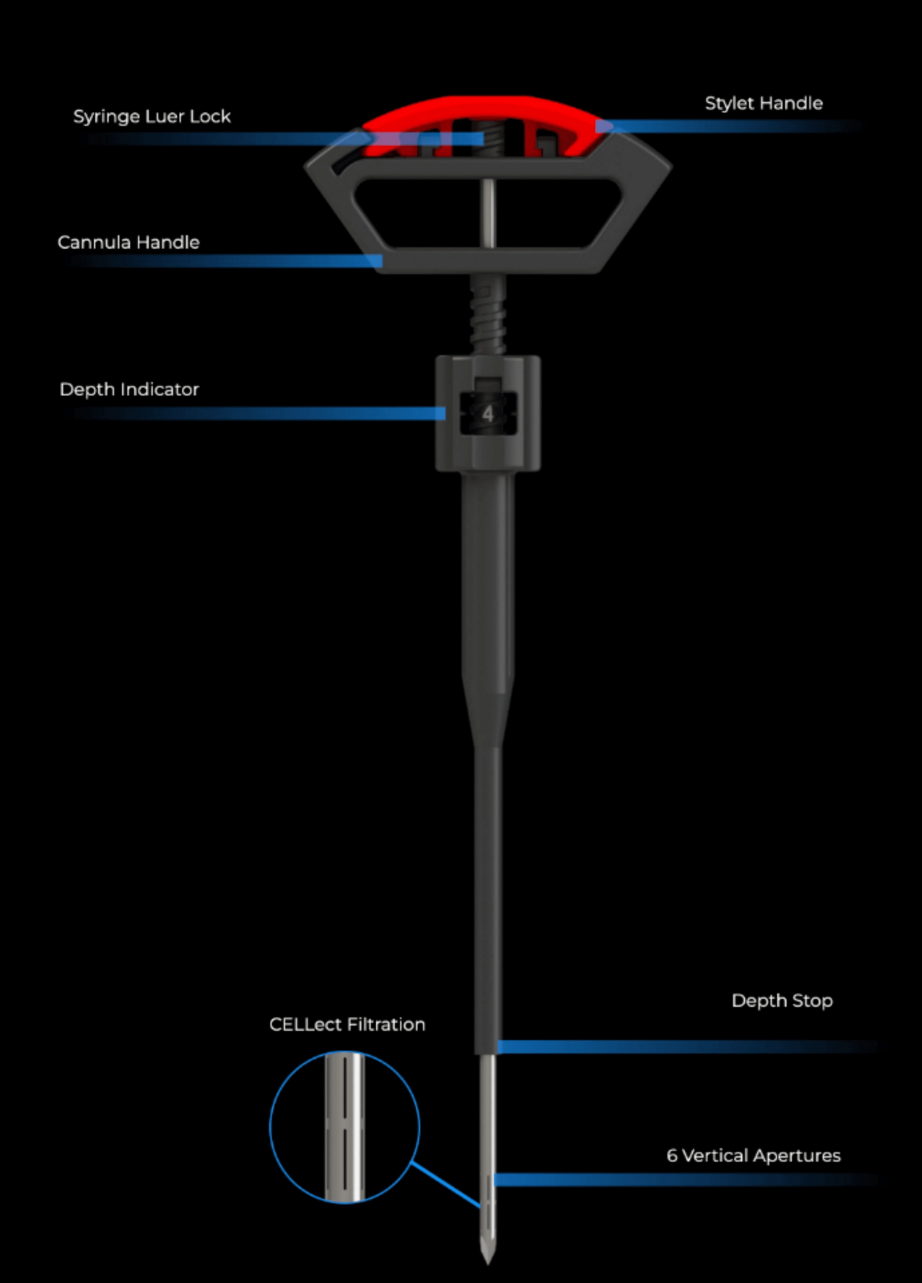
Schematic representation of the B-MAN™ bone marrow aspirate system and its labeled components Shown are the proximal syringe luer lock, stylet handle, cannula handle, adjustable depth indicator, shaft, depth stop, and the distal aspiration segment featuring CELLect filtration with six vertical apertures (magnified inset). The image is provided by and reproduced with permission from SurGenTec, LLC, the manufacturer and copyright holder of the B-MAN™ Bone Marrow Aspiration System. All rights reserved.

**Figure 2:**
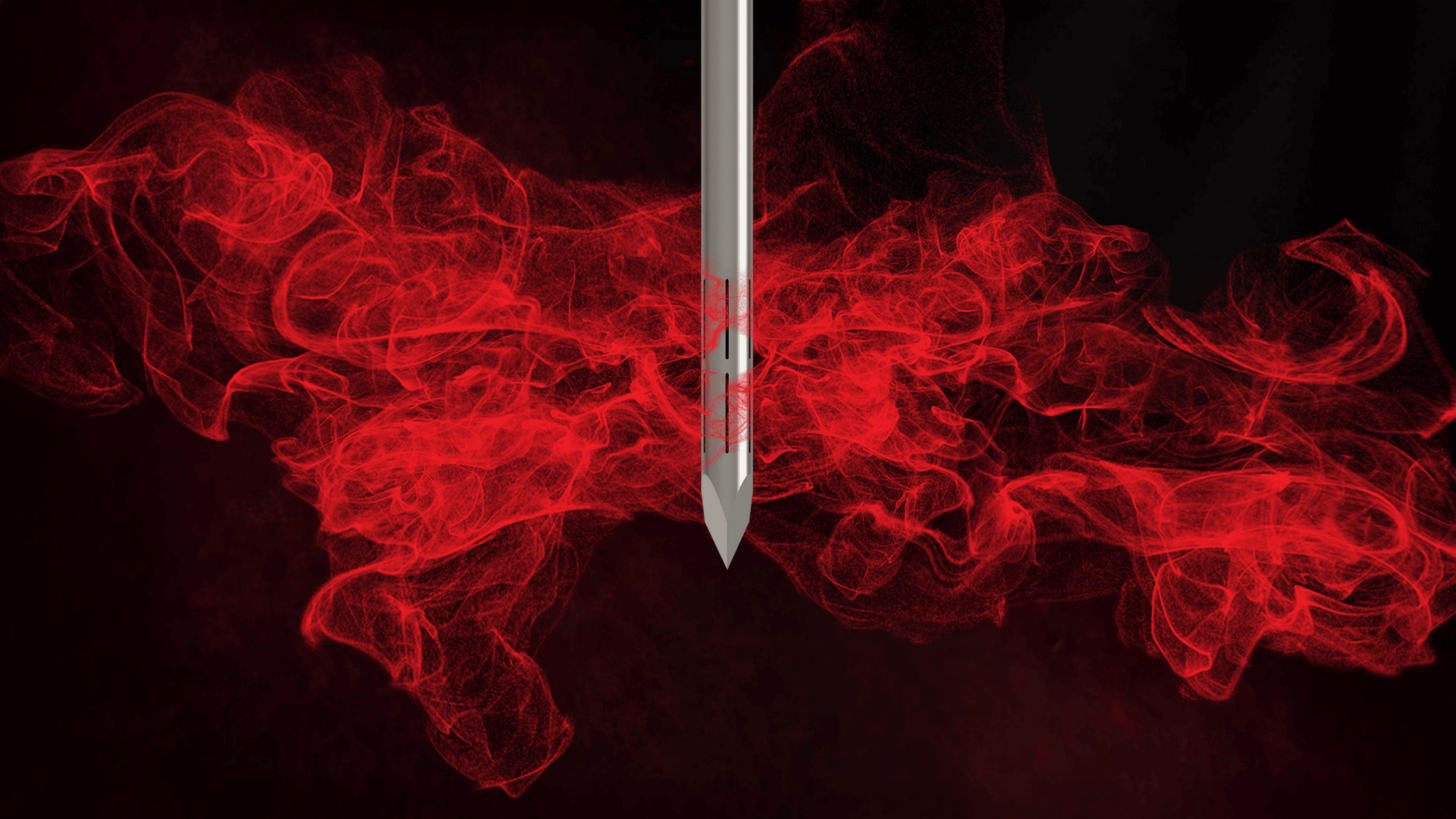
B-MAN™ graphic depicting blood flow and surface area of elongated aspirate windows allowing for extraction of bone marrow aspirate The image is provided by and reproduced with permission from SurGenTec, LLC, the manufacturer and copyright holder of the B-MAN™ bone marrow aspiration System. All rights reserved.

The B-MAN™ system incorporates a trocar designed to facilitate controlled entry into the intramedullary space. In addition, the system utilizes integrated filtration intended to reduce the presence of bone spicules and other particulate contaminants during aspiration. A key engineering feature of B-MAN™ is the incorporation of slit aspiration geometries (Figure 1). Rather than relying solely on discrete circular fenestrations, the aspiration cannula incorporates multiple longitudinal slits arranged circumferentially along the shaft. This configuration increases the open surface area for marrow flow up to 2.5 times that of comparable needles, thereby distributing negative pressure across a broader surface, consistent with the principle that harvesting technique and aspiration mechanics influence the degree of hemodilution and progenitor cell yield [1,2]. By reducing localized suction velocity at the individual intake points, the design is intended to moderate shear forces at the needle-marrow interface and support the collection of marrow-rich aspirate for a greater portion of the aspiration cycle [1,2,13]. Through the combined effects of the distributed suction geometry and aspiration strategy, the B-MAN™ system is designed to reduce peripheral blood ingress during aspiration, which is an important driver of declining progenitor concentration with increasing aspirate volume [1,2] (Figure 2).

Laboratory analysis

All samples were processed at the Department of Biology, Franciscan University of Steubenville, the same reference laboratory utilized in the previously published comparative study by Scarpone et al. [3] evaluating optimized no-spin aspiration systems and centrifuge-processed bone marrow concentrate (BMAC). The deliberate use of a similar laboratory protocol and analytical facility as this prior benchmark study was a methodological choice to support the interpretability of descriptive cross-study comparisons [10]. CFU-f was selected as a functional surrogate for mesenchymal stromal progenitor content, acknowledging known heterogeneity within this population [15].

Total nucleated cells and viability

To determine TNC levels, the bone marrow aspirate was diluted 1:20 in Hanks' Balanced Salt Solution (HBSS) and mixed 1:1 with AO/PI dye (Nexcelom Bioscience, Lawrence, MA). The sample was then counted using a Cellometer Vision CBA Cytometry System (Nexcelom Bioscience) to determine the concentration of live and dead nucleated cells. All samples were counted in duplicate.

Colony-forming unit-fibroblast assay

To measure CFU-f levels, a volume of bone marrow containing 500,000 nucleated cells was plated in a T-25 flask containing 5 mL of DMEM/F12 media (GIBCO, Thermo Fisher Scientific, MA) supplemented with 10% MSC-qualified fetal bovine serum (FBS) (GIBCO) and an antibiotic/antimycotic mix (GIBCO). Cells were cultured under standard conditions, 37°C, 5% CO₂. After 72 hours, the flasks were washed three times with HBSS to remove non-adherent cells. The cells were then cultured for 9 additional days. After 12 days total, the colonies were stained with 0.5% crystal violet solution in methanol. Colonies with 100 or more cells were counted as CFU-fs. All samples were processed in duplicate.

CD34+ enumeration by flow cytometry

The levels of CD34+ cells were determined using the established ISHAGE protocol [21,22]. Briefly, 50 µL of marrow was mixed with 47 µL of cell staining buffer, 1.5 µL of phycoerythrin (PE)-conjugated CD34 antibody (BioLegend), and 1.5 µL of fluorescein isothiocyanate (FITC)-conjugated CD45 antibody (BioLegend), and incubated for 20 minutes at room temperature. 1.4 mL of RBC lysis buffer was then added, and samples were run on an Accuri flow cytometer using gates established per the ISHAGE protocol.

Statistical considerations

Given the sample size (n = 5), results are presented as descriptive statistics (mean, range). No inferential statistical comparisons were performed between B-MAN™ results and historical benchmark data, as subjects, operators, and procedural contexts differed across studies. Cross-study comparisons are descriptive and intended to provide preliminary context for the observed values.

## Results

Across five male subjects (age range 18-44) undergoing a range of elective orthopedic procedures, unprocessed bone marrow aspirates of 1 mL were obtained using the B-MAN™ system had the following mean laboratory values: TNC concentration of 102 × 10⁶ cells/mL, colony-forming unit-fibroblast (CFU-f) concentration of 15,145 CFU-f/mL, CD34+ cell concentration of 1,143,216 cells/mL, and mean cell viability of 96.9% (Table 1). Two of five subjects exceeded the published upper bound of this range. These values represent laboratory cell concentrations at the point of harvest without centrifugation. Notable inter-subject variability was observed, with TNC values ranging from 37 to 204 × 10⁶/mL and CFU-f values ranging from 3,174 to 40,330/mL, consistent with known biologic variation in marrow cellularity across individuals [1].

**Table 1:** Patient demographics, procedures, and B-MAN™ aspirate laboratory results All subjects were male. ACL = anterior cruciate ligament reconstruction. TNC = total nucleated cells; CFU-f = colony-forming unit–fibroblast. Continuous variables are presented as Mean ± SD; categorical variables are presented as N. Group averages: Age 31.0 ± 9.3 years; TNC 102.2 ± 66.0 × 10⁶/mL; CFU-f 15,145 ± 15,131/mL; CD34⁺ 1,143,216 ± 513,767 cells/mL; viability 96.9 ± 1.4%. No inferential statistical testing was performed in this proof-of-concept analysis; descriptive statistics only. When inferential comparisons were drawn from cited studies, statistical significance was defined as p < 0.05.

Subject	Age	Sex	TNC (×10⁶/mL)	CFU-f (/mL)	CD34+ (cells/mL)	Procedure	Viability (%)
1	18	M	37	3,174	627,600	ACL	96.3
2	29	M	204	40,330	1,963,900	ACL	95.6
3	31	M	76	12,187	901,670	Hip Labrum	95.9
4	33	M	129	16,215	1,278,911	ACL Tibial Plateau	99.1
5	44	M	65	3,820	944,000	Achilles	97.5
Averages	31	—	102	15,145	1,143,216	—	96.9

Aspirate quality assessment

The frequency of CFU-fs in marrow, as a ratio of TNCs, has been reported to range from 0.001% to 0.01% [7]. The mean individual patient CFU-f:TNC ratios observed in this study was 0.013% (range 0.006-0.020%). Collectively, these ratios suggest that the aspirates were composed predominantly of marrow-derived cells with minimal peripheral blood dilution. This internal consistency metric provides independent confirmation that the observed cell yields reflect a marrow-rich aspirate rather than an artifact of processing or measurement.

Descriptive comparison with published benchmarks

When compared descriptively with published benchmark data analyzed under similar laboratory conditions, these values place B-MAN™ aspirates above reported ranges for both centrifuge-processed BMAC systems and previously published optimized no-spin aspiration devices [2-5]. Scarpone et al. compared the Marrow Cellution device, marketed as a no-centrifuge option, to Harvest and Emcyte centrifuge-based concentration systems [3]. Part of the analysis was performed in the same laboratory as the present study and evaluated the same number of patients. When compared to the Scarpone et al. results, B-MAN™ produced higher TNC counts than the Marrow Cellution device as well as the Harvest and Emcyte concentration systems (Table 2). The difference was most pronounced for CFU-f concentrations, where B-MAN™ values exceeded all comparators by a substantial margin (Table 3).

**Table 2:** Total nucleated cell concentrations: B-MAN™ vs. published comparators Data are presented as Mean total nucleated cell (TNC) concentration (× 10⁶ cells/mL); B-MAN value represents Mean ± SD of 102.2 ± 66.0 × 10⁶/mL (N = 5). Comparator values are reported means as published by Scarpone et al. [3] and are presented for descriptive benchmarking only; no inferential statistical comparison was performed between the present cohort and historical comparators. Where original between-group comparisons in the cited reference are noted, statistical significance was defined as p < 0.05.

System	TNC (×10⁶/mL)	Processing
B-MAN™ (current study)	102	None
Harvest BMAC-Comparison 1 (3)	66.1	Centrifuge
Emcyte BMAC-Comparison 3 (3)	60.5	Centrifuge
Marrow Cellution-Comparison 3 (3)	36.7	None
Marrow Cellution-Comparison 1 (3)	31.5	None

**Table 3:** Colony-forming unit–fibroblast concentrations: B-MAN™ vs. published comparators Data are presented as Mean colony-forming unit-fibroblast (CFU-f) concentration (colonies/mL); B-MAN value represents Mean ± SD of 15,145 ± 15,131/mL (N = 5). Comparator values are reported means as published by Scarpone et al. (3) and are presented for descriptive benchmarking only; no inferential statistical comparison was performed between the present cohort and historical comparators. Where original between-group comparisons in the cited reference are noted, statistical significance was defined as p < 0.05.

System	CFU-f (/mL)	Processing
B-MAN™ (current study)	15,145	None
Marrow Cellution-Comparison 3 (3)	2,263	None
Marrow Cellution-Comparison 1 (3)	1,583	None
Harvest BMAC-Comparison 1 (3)	797	Centrifuge
Emcyte BMAC-Comparison 3 (3)	267	Centrifuge

## Discussion

This laboratory evaluation demonstrates that high concentrations of progenitor cells can be obtained in unprocessed bone marrow aspirate using a novel aspiration interface. The observed laboratory yields are generally higher than values reported in published benchmarking studies evaluating optimized aspiration techniques [1-3].

Contextualizing the observed cell yields

The Scarpone et al. study compared the Marrow Cellution device to the Harvest and Emcyte centrifuge-based concentration systems, with all CFU-f analyses performed in the laboratory used in the present investigation and with the same number of patients [3]. It should be noted that the present aspirations were obtained from the anterior iliac crest, whereas the Scarpone et al. benchmark cohorts utilized the posterior iliac crest [3,8]. Although both sites are validated marrow sources for orthopedic biologics, donor-site differences in cellularity have been described and should be considered when interpreting the descriptive cross-study comparison. When the present results are compared with the benchmark data, B-MAN produced higher TNC counts and fold increases in CFU-f in the Marrow Cellution device, as well as in the Harvest and Emcyte concentration systems (Tables 2, 3). The CFU-f difference is particularly notable: the observed mean of 15,145 CFU-f/mL represents a 6.7-fold increase over the highest Marrow Cellution value (2,263/mL), a 19-fold increase over Harvest BMAC (797/mL), and a 57-fold increase over Emcyte BMAC (267/mL). These concentrations are also substantially higher than those reported in dose-escalation analyses of BMAC therapy for knee osteoarthritis [18]. The convergence of similar protocols and the use of the same analytical facility and SOPs strengthens the interpretability of these descriptive comparisons, with the anatomical-site difference noted above as an acknowledged caveat [3]. Because no concurrent controls were used in the present study, these comparisons should be considered hypothesis-generating rather than confirmatory of device superiority.

Interestingly, Hernigou’s study on small volumes and syringe size evaluated 1 mL draws from 20 patients with a 10 mL syringe. Although lab analysis conditions were different from those of this study, when compared to these results, B-MAN yielded a 7-fold increase in CFUs. The CFU-f:TNC ratio offers a particularly informative lens through which to evaluate aspirate quality, because it moves in a predictable direction depending on the composition of the sample. In a hemodiluted aspirate, one contaminated with peripheral blood, the denominator (TNC) rises rapidly as blood-derived nucleated cells flood the sample, while the numerator (CFU-f) remains relatively flat because peripheral blood contains very few colony-forming progenitors [1,8]. The result is a ratio that falls well below published norms. This is the signature of a blood-dominant aspirate and the central problem that aspiration technique seeks to avoid.

The opposite pattern was observed in the present study. The mean of individual patient CFU-f:TNC ratios was 0.013% (range 0.006-0.020%), calculated as the average of each subject’s CFU-f/TNC ratio. Published literature reports this ratio at 0.001-0.01% in minimally diluted marrow [7]. Three of five subjects fell within the published range, and two were slightly above this range. This pattern is inconsistent with peripheral blood contamination and instead suggests that the B-MAN system produced aspirates enriched in marrow-derived progenitor content relative to total cellularity. The modest exceedance of the published range is consistent with known inter-subject variability in marrow cellularity [1,8] and may reflect the capacity of this novel aspiration geometry to preferentially mobilize progenitor-rich marrow while limiting the influx of progenitor-poor peripheral blood.

The CD34+ cell counts observed in this study provide additional context regarding the hematopoietic component of the aspirate. The mean CD34+ concentration of 1,143,216 cells/mL, with a range of 627,600 to 1,963,900 cells/mL, is consistent with the overall pattern of high cellular yield observed across TNC and CFU-f metrics. CD34+ cells represent hematopoietic and endothelial progenitor populations and are commonly used as a marker of bone marrow-derived cellularity [3,8]. While direct cross-study comparisons of CD34+ concentrations are limited by known differences in flow cytometry protocols, gating strategies, and reporting methodologies [3], the magnitude and variability of the values observed in the present study are consistent with a marrow-rich aspirate rather than a hemodiluted, blood-dominant sample [1,8]. The parallel elevation of CD34+, CFU-f, and TNC concentrations further supports the interpretation that the aspirates retained a broad representation of marrow-derived cellular compartments at the point of harvest [1,3,8].

The observed CFU-f:TNC ratios warrant additional consideration. Published estimates of this ratio in minimally diluted marrow have been reported to range from approximately 0.001% to 0.01% [7]; however, the mean ratio in the present study was 0.013%, with two of five subjects exceeding the upper bound of this range. This finding should not be interpreted as confirmatory in isolation, but rather as a deviation requiring contextual interpretation. Several factors may contribute to this observation, including inter-subject variability in marrow cellularity [1,8], differences in CFU-f assay sensitivity and colony-identification thresholds [3], and potential variability in total nucleated cell enumeration [3]. In addition, the reference range cited from earlier studies may not be directly comparable to the present assay conditions, particularly given known methodological differences in plating density, culture conditions, and colony definition criteria across studies [3,7]. Importantly, the directionality of the ratio remains inconsistent with hemodilution, which is typically characterized by a reduction in CFU-f:TNC values due to disproportionate increases in blood-derived nucleated cells [1,8]. As such, while the elevated ratios observed in a subset of subjects should be interpreted cautiously, they do not contradict the broader finding that the aspirates demonstrated high progenitor cell content at the point of harvest.

Mechanistic considerations

These findings support the hypothesis that modifying the aspiration interface geometry may complement established strategies that focus primarily on needle tip relocation and aliquot control. The well-established benefits of small-aliquot aspiration are supported by the foundational work of Muschler et al. [1] and Hernigou et al. [2], who demonstrated that limiting aspirate volume to 1-2 mL per site and using smaller syringes with rapid plunger displacement significantly improve progenitor yield.

However, to obtain 8-10 mL aspirates with the same high progenitor cell count, using a needle with slit aspiration geometry appears beneficial. Traditional circular fenestrations create localized high-velocity inflow that can generate shear forces sufficient to disrupt sinusoidal vessels [13]. By distributing negative pressure across a larger surface area through longitudinal slits, the B-MAN design may reduce peak local velocity and shear stress at the marrow interface. This principle is analogous, by way of design analogy and pending direct computational fluid-dynamics validation in the context of marrow aspiration, to findings in hemodialysis catheter design, where side-hole geometry has been shown to influence flow characteristics and performance [13]. In the marrow aspiration context, reduced localized shear may help preserve sinusoidal integrity, thereby delaying the onset of peripheral blood contamination and extending the window during which marrow-rich aspirate can be collected. By moderating local inflow dynamics, incorporating slit aspiration geometries may also reduce technique sensitivity and contribute to more reproducible aspirate quality across operators [1,2].

Procedural efficiency and workflow implications

Although this study did not formally measure procedural time, the workflow differences between the B-MAN™ system and centrifuge-based concentration are substantial and merit consideration in the context of operating room efficiency. Centrifuge-based BMAC workflows involve multiple sequential steps. First, 60-120 mL of bone marrow must be aspirated using best practices, typically requiring repeated needle repositioning over up to 5-10 minutes to minimize hemodilution at each site [1,2]. The aspirate is then removed from the sterile field and transferred to the centrifuge system, where processing requires one or more spin cycles [14]. The aspirate is then returned to the sterile field for application. In practice, total centrifuge processing time is commonly reported at 20-30 minutes. When aspiration and processing are combined, the total time from cortical access to a ready-to-inject BMAC product is estimated at 30-45 minutes in most published workflows.

**Figure 3:**
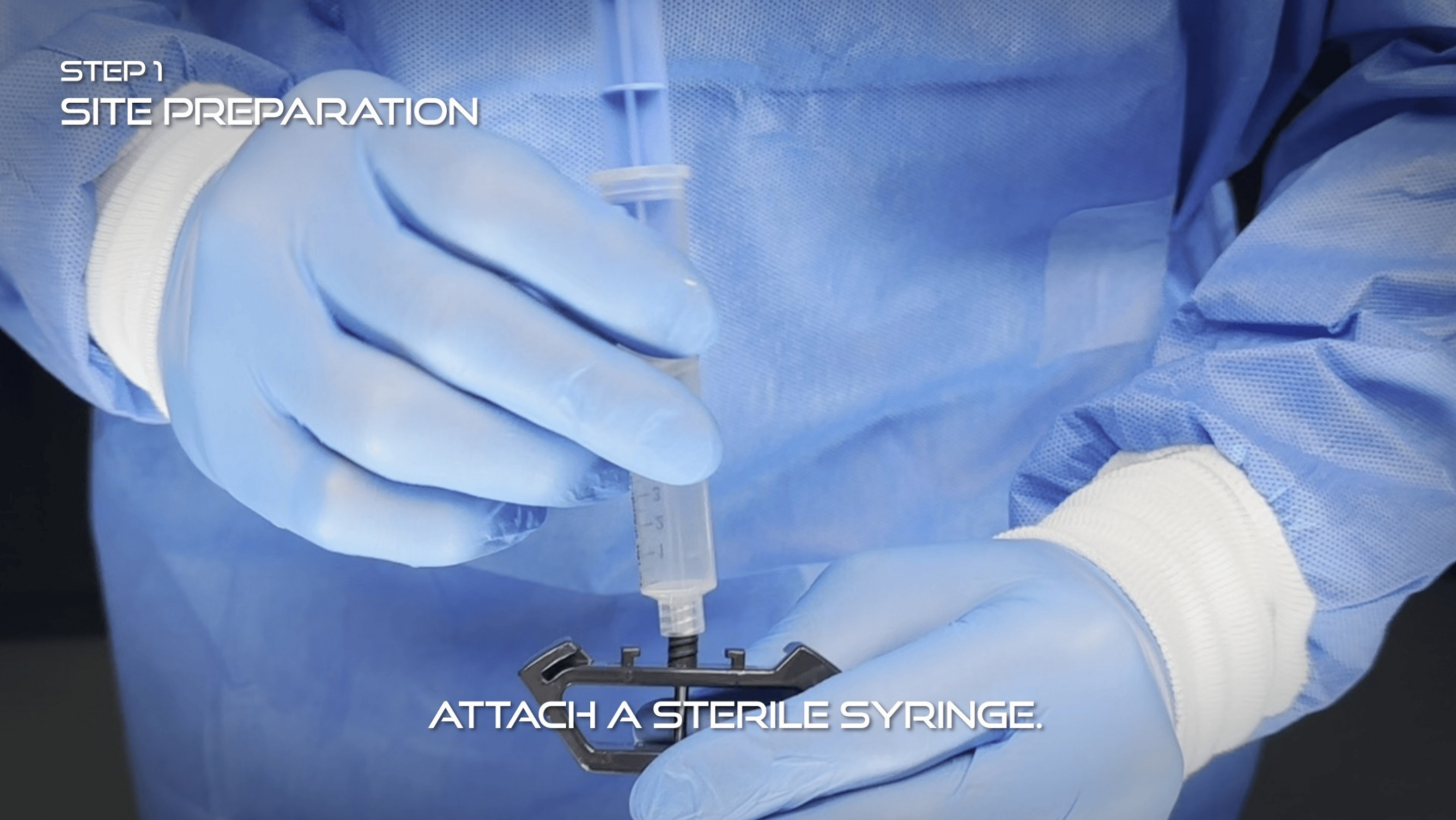
Illustration of the B-MAN™ bone marrow aspiration system, depicting the syringe/luer lock engagement The image provided by and reproduced with permission from SurGenTec, LLC, the manufacturer and copyright holder of the B-MAN™ Bone Marrow Aspiration System. All rights reserved.

By contrast, bone marrow aspiration using the B-MAN system is reported to take approximately 90 seconds from cortical access to injection, with no post-harvest processing required (Figure 3). The aspirate is used in its native form, eliminating centrifugation, sample transfer, and the associated equipment and personnel requirements.

The volume differential further underscores the efficiency of the B-MAN™ approach. Centrifuge-based systems require 60-120 mL of aspirate as starting material, volumes that inherently increase the degree of hemodilution, as progenitor concentration declines with each successive milliliter drawn from a fixed site [1,2,9]. This large-volume requirement is not incidental; it is necessary because centrifugation can only concentrate what is already present in the aspirate, and a hemodiluted starting sample yields a hemodiluted concentrate regardless of fold increase [4]. By contrast, the B-MAN™ system requires no more aspirate per subject than is required for the desired application, typically not more than 7-10 mL, yet this small comparative volume, without any post-harvest processing, may yield TNC and CFU-f concentrations that exceed those achieved by centrifuge systems processing 6 to 12 times the volume.

In addition, the procedural time difference, estimated at approximately 30-43 minutes of saved operating room time, carries meaningful economic implications. Published estimates of operating room costs range from $36-$46 per minute in the United States [20], with some analyses reporting costs exceeding $60 per minute for high-complexity cases. At these rates, a 30-minute reduction in OR time would yield estimated savings of $1,100-$1,800 or more per case, exclusive of the capital and disposable costs associated with centrifuge equipment. As a consequence, the elimination of centrifugation from the bone marrow augmentation workflow could represent a meaningful improvement in both clinical efficiency and healthcare value.

Like the B-MAN™, the Marrow Cellution system evaluated by Scarpone et al. utilizes a small-volume aspiration approach to limit hemodilution [3]. However, the B-MAN™ produced a mean CFU-f concentration approximately 6.7-fold higher than the highest Marrow Cellution value. The difference in progenitor yield suggests that the aspiration interface geometry is a key differentiating variable and reinforces the principle that aspirate quality is affected by how it is collected, specifically, by the fluid dynamics at the needle-marrow interface [1-3].

Relationship to clinical outcomes

Although prior studies have suggested associations between progenitor cell dose and healing in select orthopedic contexts, including nonunion and spinal fusion, such relationships are indication-specific and cannot be inferred from laboratory yield metrics alone [4,5]. Hernigou et al. demonstrated that successful nonunion healing was associated with graft CFU-f concentrations exceeding 1,500 progenitors/cm³, while patients who failed to unite received significantly fewer progenitors [4,18]. The mean CFU-f concentration observed in the present study (15,145/mL) is approximately 10-fold above this threshold. Similarly, Chaput et al. reported correlations between marrow cellular composition and spinal fusion outcomes, emphasizing the importance of both progenitor concentration and total cell delivery [5,18]. However, no clinical outcome claims can be made based on the present findings. Prospective clinical trials are required to determine whether these in vitro yields translate to improved healing rates across specific orthopedic applications.

Limitations

This study has several noted limitations. The sample size of five subjects is small, limiting the generalizability of the findings. All subjects were male, so the results may not be representative of female patients, in whom marrow cellularity may differ. The study employed a non-randomized, retrospective design without concurrent controls; comparisons with published benchmark data are descriptive and cross-study in nature, and definitive conclusions regarding device superiority cannot be drawn. Only laboratory cell-yield metrics were evaluated; no clinical outcomes, procedural times, or patient-reported outcomes were assessed. Aspirations were limited to a single anatomical site (anterior iliac crest); performance at other sites has not been characterized. All aspirations were performed by experienced orthopedic surgeons, and formal inter-operator variability was not assessed, which is a planned focus of future multi-site work.

## Conclusions

In this descriptive laboratory evaluation, a bone marrow aspiration system incorporating thin, elongated intake geometries yielded high concentrations of total nucleated cells, CFU-f, and CD34+ cells with high viability in unprocessed aspirate. These results place B-MAN aspirates at the upper range of values reported in published benchmark studies of both centrifuge-processed BMAC systems and optimized no-spin aspiration devices. The findings support further investigation of aspiration interface engineering as a complementary approach to reducing hemodilution and improving marrow aspirate quality. Prospective clinical studies with larger sample sizes and concurrent controls are required to determine the reproducibility and clinical relevance of these findings across orthopedic applications. Future studies are planned to evaluate head-to-head comparisons with BMAC, the effect of larger sample volumes, and clinical evaluation in various surgical applications.
